# Periapical Status and Quality of Root Canal Fillings in a Moroccan Subpopulation

**DOI:** 10.1155/2017/1068982

**Published:** 2017-06-21

**Authors:** Hafsa El Merini, Hind Amarir, Amine Lamzawaq, Mouna Hamza

**Affiliations:** ^1^Department of Conservative Dentistry and Endodontics, Faculty of Dentistry, University Hassan II Casablanca, Casablanca, Morocco; ^2^Private Practice, Casablanca, Morocco; ^3^Department of Statistics, Faculty of Dentistry, University Hassan II Casablanca, Casablanca, Morocco

## Abstract

**Aim:**

The aim of the study was to assess the prevalence of apical periodontitis (AP) and quality of root canal fillings in an adult Moroccan subpopulation.

**Methods:**

In the study 508 patients were included, attending the Conservative Dentistry Clinic at the Faculty of Dentistry of Casablanca. 508 panoramic and 709 periapical radiographs were observed. The periapical status of all teeth (with the exception of third molars) was examined according to Ørstavik's periapical index. The statistical analysis was performed with the software Epi Info Version 6.04dfr, April 2001.

**Results:**

A total of 12719 teeth were examined. 45.3% of patients had apical periodontitis. 4.2% of teeth were treated endodontically and 70.4% of these treatments were inadequate. 91,5% with inadequate endodontic treatment presented apical periodontitis, while only 8,2% with adequate endodontic treatment had apical periodontitis. The lower molars and the upper premolars were the most affected teeth. The presence of apical periodontitis was correlated significantly with insufficient root canal fillings (*p* < 0.05).

**Conclusions:**

The present study found a high prevalence of apical periodontitis in this Moroccan population. Inadequate root canal fillings were associated with an increased prevalence of apical periodontitis.

## 1. Introduction

Apical periodontitis (AP) is an inflammation disease of periradicular tissues; it occurs as a result of various insults to the dental pulp, including caries and physical and iatrogenic trauma or after a failed endodontic treatment [1–3].

The diagnostic of AP is based on the presence of symptoms and clinical signs during clinical examination and analysis of radiographs (periapical or panoramic). Radiographic analysis is important, because AP in its chronic form is often asymptomatic and left untreated [4].

Endodontic epidemiological studies are necessary because they allow knowing the distribution and the prevalence of AP and its determinants, including treatment outcome in the studied populations which are evaluated by the presence or absence of AP [5].

Several cross-sectional studies have presented data regarding the prevalence of AP and endodontically treated teeth; these data vary depending on the population studied, the radiographic methods, and the classifications of AP used [6–8].

Data on the prevalence and quality of root canal fillings are rare in Morocco. Considering the fact that these data are important for the evaluation of the quality of the endodontic treatment realized as well as for dental care planning and the need of such information, the aim of this study was to investigate the prevalence and quality of root canal fillings and their association with periapical periodontitis in a group of patients from Morocco.

## 2. Materials and Methods

### 2.1. Study Population

The sample in this cross-sectional study was formed of patient records. Patients were aged between 10 and 60, attending routine dental treatment (not emergency care) at the University of Casablanca, Faculty of Dentistry, for the first time during the period of May 19, 2014, to June 20, 2014.

### 2.2. Data Collection

Data were collected retrospectively from the patients' medical records, which include all the information needed for the study, the medical history, and a complete examination of the oral cavity. The data also include panoramic and periapical radiographs.

The information required for our study was recorded in a data collection sheet, including patient name, medical record number, sex, age, missing teeth, nonrestored teeth, fillings, crowns, endodontically treated teeth, and teeth with apical periodontitis.

### 2.3. Radiographic Evaluation

Teeth were classified as endodontically treated if a radiopaque material was detected in the pulp chamber and/or in one or more of the root canals. Root fillings were classified as adequate or inadequate on the basis of guidelines published by the European Society of Endodontology (ESE) [9]; the technical quality of the root fillings was evaluated according to length from the root apex and homogeneity (adaptation to the canal walls, voids, and porosities). Indeed, the root canal filling was assessed using the following criteria:

- Adequate: filling material is present in the root canal within 0–2 mm of the radiographic apex. Proper density is absence of lateral spaces along root canals or voids.
- Inadequate: root canal fillings end is more than 2 mm short of the radiographic apex, or extruded beyond the apex, or present only in the pulp chamber. Inadequate also means presence of lateral spaces or voids, inadequate density, unfilled canals, and/or poor condensation.

The periapical health was assessed using the periapical index proposed by Ørstavik et al. [10] in 1986, who scored the apical area of the radiographic images as follows: (1) normal periapical structures; (2) small changes in bone structure; (3) changes in the bone structure with little mineral loss; (4) periodontitis with well-defined radiolucent area; (5) severe periodontitis with exacerbating features. Scores (1) and (2) represented healthy periapical status, while (3), (4), and (5) are signs of AP. Multirooted teeth were classified according to the root with the most severe periapical status.

The parameters used in this study (overall quality of root filling based on length/density of root filling and presence/absence and the degree of AP) were evaluated based on those described by other researchers [11–13].

The radiographs were analyzed by two experienced and calibrated examiners using an illuminated viewer box and magnification (3,5x).

### 2.4. Statistical Analysis

After data collection, statistical analysis was performed using Epi Info Version 6.04dfr software. AP was calculated regarding the total number of individuals, the total number of teeth, location, tooth group, and endodontic treatment. Significance level was set at *p* < 0.05.

## 3. Results

In a period of one month, from May 19, 2014, to June 20, 2014, a total of 508 panoramic and 709 periapical radiographs were examined, and 12719 teeth were observed.

The sample for this cross-sectional study consisted of 508 patients, with 269 females (53%) and 239 males (47%).

From clinical records and X-rays, it was found that the total number of nonrestored teeth was 342 teeth (2.7%), 687 (5.4%) with coronal restorations, and only 42 (0.3%) crowned (Table 1).

Endodontic treatment was detected in 537 (4,2%); the upper premolars had the highest rate with 140 treated teeth (26%), whereas the teeth with the lowest prevalence were the mandibular incisors with 4 teeth (0.7%) (Table 1).

Among the 508 patients in the study population, 230 (45.3%) had apical periodontitis. AP was observed in 526 teeth (4%). The most affected were the lower molars with 133 teeth, the upper premolars with 111 teeth, and the upper molars with 88 teeth (25.4%, 21.1%, and 16.7% resp.) (Table 1).

The total number of teeth with adequate endodontic treatment was 159 (29.6%); inadequate endodontic treatment was observed in 378 (70.4%).

13 teeth with adequate treatment (8.2%) had AP, and 346 teeth with inadequate endodontic treatment (91.5%) had AP (Table 2).

AP was detected in 359 endodontically treated teeth (66.8% of treated teeth); the upper premolars were the most frequent AP affected root-filled teeth (25.3%) (Table 3).

## 4. Discussion

Chronic, apical periodontitis is an inflammation of the periodontium; it occurs as a result of pulp necrosis and is often discovered as incidental findings on radiographic survey [1, 3, 14].

A total of 508 panoramic and 709 periapical radiographs were examined. The periapical radiographs were systematically performed whenever a panoramic X-ray showed a tooth, a coronary, or radicular obturation with suspicion of a periapical lesion. The records of patients, whose radiographic examinations could not be made, regardless of the cause, were excluded from the study. Although panoramic radiographs provide a general overview of dentomaxilomandibular structures, periapical radiographs had higher definition and yielded more information on the status of the periapical bone [15], so periapical radiographs were used in this study mainly to evaluate the degrees of apical periodontitis and the quality of the endodontic treatments.

Previous studies had already used the same techniques for the same reasons [16–20]. Other epidemiological studies [21, 22] concluded that the use of panoramic radiography in epidemiologic studies of dental health was acceptable; Muhammed and Manson-Hing [23] found no statistically significant difference between panoramic and periapical radiographs in the detection of AP.

Recently, a new imaging modality, cone beam computed tomography (CBCT), has been shown to be a useful tool in a number of endodontic applications. This 3-dimensional imaging technique allows for a more precise diagnosis of periapical lesions [24]. However, the higher effective dose of ionizing radiation compared to conventional 2-dimensional radiographs is rarely justified. Some scientists have suggested the use of CBCT when conducting future research, in order to minimize the technical limitations of conventional radiographs, especially when new scanners with lower radiation doses are available [25, 26].

In order to evaluate the periapical status, the periapical index (PAI) developed by Ørstavik et al. [10] was adopted. PAI is a scoring system for radiographic assessment of apical periodontitis which presents good accuracy and reproducibility [27].

The sample contained more females (53%) than males (47%). This difference is not significant except the fact that women are more healthcare conscious than males and have a greater interest in receiving dental care. Other studies in this field found a similar gender proportion [18, 28–30].

In a sample of 508 patients, 230 (45.3%) had apical periodontitis. This prevalence is lower than that found by Chala et al. (63.79%) [31], which was the only study carried out in Morocco, at dental clinic of Rabat University. The prevalence found is higher than that reported by Georgopoulou et al. (13,6%) [29] and Loftus et al. (33%) [28]. On the other hand, it remains lower than that found by Boucher et al. (63%) [16], Jiménez-Pinzón et al. (61%) [18], and De Moor et al. (73%) [32]. This difference in the prevalence of apical periodontitis observed between our study and other studies could be explained by the difference in methodology and difference in oral health policies between countries.

526 teeth (4.1%) had apical periodontitis, the most affected teeth were the lower molars (25.4%), which corroborates the results of Peters et al. [33], who found that the highest prevalence of apical periodontitis occurred in the second mandibular molars (16.9%), followed by the first mandibular molars (13%).

537 teeth (4.2%) had undergone root canal treatment, with a ratio of one tooth being treated endodontically per patient. This rate is lower than that found by Matijević et al. (8.5%) [11], Ilić et al. (12.5%) [12], and Boucher et al. (19%) [16], almost similar to that declared by Peters et al. (4.8%) [33], and higher than that found by Jiménez-Pinzón et al. (2.4%) [18].

The upper premolars are the most frequently treated teeth in our survey. The distribution of root canal treatment according to the tooth type was analyzed in many studies; the findings are very diverse [12].

The relationship between the quality of endodontic treatment and the prevalence of apical periodontitis has been demonstrated in a large number of epidemiological studies [11, 16, 34–37]. The present work showed that more than two-thirds of root canal-treated teeth (66.8%) presented apical periodontitis. This rate is higher than that found by Chala et al. in Morocco (39.5%) [31] and other studies in Saudi Arabia (58.6%) [34], France (33%) [13], Brazil (16.70%) [38], and Palestine 59.5% [39].

Quality of root canal filling is one of the keys of the success of endodontic treatment. In our study, same as in other comparable studies, the quality of the root canal filling is estimated by two criteria: the length of the root canal filling from the radiographic apex (0–2 mm) and the appearance of the canal obturation in terms of density and tightness. The results of the present study showed that only 29.6% of endodontic treatments were adequate, which confirmed the results of other surveys that showed that a large percentage of root canal fillings were not acceptable [32, 40, 41].

High frequency of AP in teeth with inadequate root canal fillings has been reported in several previous studies [27, 36, 42, 43]. In this study, inadequate treatments were associated with a significantly high prevalence of periodontitis; 91.5% of inadequate endodontic treatments had apical periodontitis and only 8.5% of the appropriate treatments presented AP.

## 5. Conclusion

The frequency of AP in endodontically treated teeth of this patient group was high, especially in teeth with inadequate root fillings, demonstrating that the quality of endodontic treatment is a factor associated with the appearance of apical periodontitis.

## Figures and Tables

**Table 1:** Distribution of missing teeth, nonrestored teeth, fillings, crowns, endodontically treated teeth, and teeth with apical periodontitis according to tooth type.

Tooth group	Missing	Nonrestored	Fillings	Crowns	Endodontically treated teeth	Apical periodontitis
Maxilla						
Incisors	161 (5%)	18 (5.3%)	84 (12.2%)	17 (41%)	96 (17.9%)	75 (14.2%)
Canines	124 (3.8%)	25 (7.3%)	37 (5.4%)	7 (16.7%)	39 (7.4%)	31 (5.9%)
Premolars	388 (12%)	71 (20.8%)	156 (22.7%)	6 (14.8%)	140 (26%)	111 (21.1%)
Molars	1070 (33%)	81 (23.7%)	165 (24%)	3 (7.3%)	94 (17.5%)	88 (16.7%)
Mandible						
Incisors	80 (2.4%)	8 (2.3%)	6 (0.9%)	2 (5%)	4 (0.7%)	9 (1.7%)
Canines	56 (1.8%)	17 (5%)	11 (1.6%)	1 (2.9%)	14 (2.6%)	18 (3.4%)
Premolars	340 (10.6%)	55 (16%)	76 (11%)	2 (5%)	62 (11.5%)	61 (11.6%)
Molars	1019 (31.4%)	67 (19.6%)	152 (22.2%)	3 (7.3%)	88 (16.4%)	133 (25.4%)

Total	3238 (100%)	342 (100%)	687 (100%)	42 (100%)	537 (100%)	526 (100%)

**Table 2:** Distribution of apical periodontitis according to the quality of endodontic treatment.

Endodontic treatment	Healthy periodontium	Apical periodontitis	Total
	(PAI = 1)	(PAI = 2 to 5)	
Adequate	146 (91.8%)	13 (8.2%)	159 (100%)
Inadequate	32 (8.5%)	346 (91.5%)	378 (100%)

**Table 3:** Distribution of apical periodontitis and healthy periodontium according to the tooth type with endodontic treatment.

Tooth type	Number of teeth with endodontic treatment		Healthy periodontium		Apical periodontitis	
			(PAI = 1)		(PAI = 2 to 5)	
Maxilla						
Incisors	96	(17.4%)	36	(20.2%)	60	(16.7%)
Canines	39	(7.4%)	17	(9.6%)	22	(6.2%)
Premolars	140	(26%)	49	(27.5%)	91	(25.3%)
Molars	94	(17.5%)	30	(16.8%)	64	(17.8%)
Mandible						
Incisors	4	(0.7%)	1	(0.6%)	3	(0.8%)
Canines	14	(2.6%)	5	(2.8%)	9	(2.5%)
Premolars	62	(11.5%)	17	(9.6%)	45	(12.2%)
Molars	88	(16.4%)	23	(12.9%)	65	(18.8%)

Total	537	(100%)	178	(100%)	359	(100%)
